# MODISTools – downloading and processing MODIS remotely sensed data in R

**DOI:** 10.1002/ece3.1273

**Published:** 2014-12-02

**Authors:** Sean L Tuck, Helen RP Phillips, Rogier E Hintzen, Jörn PW Scharlemann, Andy Purvis, Lawrence N Hudson

**Affiliations:** 1 Department of Plant Sciences, University of OxfordOxford, OX1 3RB, U.K; 2 Department of Life Sciences, Imperial College London, Silwood ParkBuckhurst Road, Ascot, Berkshire, SL5 7PY, U.K; 3 Department of Life Sciences, Natural History MuseumCromwell Road, London, SW7 5BD, U.K; 4 School of Life Sciences, University of SussexBrighton, BN1 9QG, U.K

**Keywords:** Conservation biology, earth observation, global change, land processes, macroecology, PREDICTS, remote-sensing, satellite imagery

## Abstract

Remotely sensed data – available at medium to high resolution across global spatial and temporal scales – are a valuable resource for ecologists. In particular, products from NASA's MODerate-resolution Imaging Spectroradiometer (MODIS), providing twice-daily global coverage, have been widely used for ecological applications. We present *MODISTools*, an R package designed to improve the accessing, downloading, and processing of remotely sensed MODIS data. *MODISTools* automates the process of data downloading and processing from any number of locations, time periods, and MODIS products. This automation reduces the risk of human error, and the researcher effort required compared to manual per-location downloads. The package will be particularly useful for ecological studies that include multiple sites, such as meta-analyses, observation networks, and globally distributed experiments. We give examples of the simple, reproducible workflow that *MODISTools* provides and of the checks that are carried out in the process. The end product is in a format that is amenable to statistical modeling. We analyzed the relationship between species richness across multiple higher taxa observed at 526 sites in temperate forests and vegetation indices, measures of aboveground net primary productivity. We downloaded MODIS derived vegetation index time series for each location where the species richness had been sampled, and summarized the data into three measures: maximum time-series value, temporal mean, and temporal variability. On average, species richness covaried positively with our vegetation index measures. Different higher taxa show different positive relationships with vegetation indices. Models had high *R*^2^ values, suggesting higher taxon identity and a gradient of vegetation index together explain most of the variation in species richness in our data. *MODISTools* can be used on Windows, Mac, and Linux platforms, and is available from CRAN and GitHub (https://github.com/seantuck12/MODISTools).

## Introduction

### Remote-sensing and ecology

Remotely sensed data – available at global scales at a fine to medium resolution, and often for free – are increasingly being used in ecology (Donoghue 2002; Kerr and Ostrovsky 2003; Bai et al. 2008; Chawla et al. 2012; Naeem et al. 2012; Sutherland et al. 2013). In particular, products derived from NASA's MODerate-resolution Imaging Spectroradiometer (MODIS) instruments (Justice et al. 1998) are a unique resource for research in many areas of ecology, conservation biology, and global change research (Table 1). Remote-sensing instruments measure physical, geochemical, and biological processes that allow us to evaluate the environment in a scalable way: from characterizing the distribution of a species or phenology of a plant community, to recording land cover change, natural or human-caused, from the ecosystem up to the global level. Several MODIS data products have been used to investigate land surface temperatures, monitor intensification of croplands, estimate volume of timber stocks, and assess change in the magnitude and frequency of fires (Table 1). Whilst it is beyond our scope to review in detail all ecological questions to which MODIS data have been applied, we discuss the relevance of MODIS data to monitoring global biodiversity change as this will be pertinent to our example analysis.

**Table 1:** Moderate-resolution Imaging Spectroradiometer products available for download using *MODISTools*, and examples of their use. Potential summary measures are techniques that are currently used in the literature and may be incorporated into *MODISTools* in future releases. Several other methods have been proposed to summarize and thereby reduce the serial correlation in time series of remotely sensed data, including principal components analysis (PCA; (Eastman and Fulk 1993), temporal Fourier processing (e.g., Scharlemann et al. 2008), and simple metrics such as bioclimatic variables (e.g., BIOCLIM variables; Xu and Hutchinson 2011)

Code	Product	Examples of product use	Potential summary measures
MOD09/MYD09	Surface reflectance	Spatiotemporal distribution of rice phenology (Sakamoto et al. 2006)	NA – reflectance data from which many measures can be derived
		Monitoring intensification of croplands (Galford et al. 2008)	
		Land cover mapping of Germany (Colditz et al. 2011)	
MOD11/MYD11	Surface temperature and emissivity	Investigating the relationship between land surface temperature and vapor pressure deficit (Hashimoto et al. 2008)	Degree days; length of period above temperature threshold
		Calculating air surface temperature using remotely sensed data and meteorological data (Benali et al. 2012)	
MOD43/MCD43	Nadir BRDF-adjusted Reflectance	Land cover mapping of South Africa (Colditz et al. 2011)	NA – reflectance data from which many measures can be derived
		Studying vegetation phenology in the United States (Zhang et al. 2003)	
MOD13/MYD13	Vegetation Indices	Crop-related LULC classification in the U.S. Central Great Plains (Wardlow et al. 2007)	Phenological measures (eg. season shift index); change vector magnitude; integrated vegetation indices
		GPP estimates for biomes across the conterminous US (Xiao et al. 2010)	
		Quantifying tree cover in an African grass savanna (Gaughan et al. 2013)	
MOD15	LAI/FPAR	Comparison of products suitable for vegetation phenology (Ahl et al. 2006)	Phenological metrics; annually integrated LAI/FPAR
		GPP estimates for biomes across the conterminous US (Xiao et al. 2010)	
MOD17	Gross primary productivity (GPP), Net photosynthesis	Calculating global terrestrial net primary production (Running et al. 2004)	Total productivity; peak NPP; seasonality of GPP
		Validation of NPP/GPP across multiple biomes (Turner et al. 2006)	
MCD12	Land cover and change	Estimation of timber volume (Nelson et al. 2009)	*Already functional in MODISTools*
		Presentation and validation of global land cover types (Friedl et al. 2002)	

Global biodiversity is declining (Balmford et al. 2003; Gaston et al. 2003; Collen et al. 2009; Tittensor et al. in press) and is predicted to continue to decline at unprecedented rates (Pereira et al. 2010; Tittensor et al. in press). Tackling this problem requires global biodiversity monitoring, such as (Collen et al. 2009; Pereira et al. 2010) which have analyzed indicators of global biodiversity change over recent decades. At global spatial scales, the time and cost involved make field-based monitoring near impossible. Remotely sensed data with global coverage and continuous, frequent measurements over periods exceeding the lifespan of most ecological studies can help bridge this gap in biodiversity monitoring; consequently, there has been an increase in the use of remotely sensed data to project biodiversity over large extents (Fuller et al. 1998; Kerr and Ostrovsky 2003; Turner et al. 2003; Pettorelli et al. 2005; Lassau and Hochuli 2008; St-Louis et al. 2014). Combining remotely sensed data with biodiversity observations using meta-analysis (Gibson et al. 2011) or synthetic analysis (Newbold et al. 2014) has become more fruitful with advances in remote-sensing instruments, data archives, and error correction methods such as the bidirectional reflectance distribution function (BRDF) that removes directional effects of view angle and illumination (Nicodemus et al. 1977). Importantly, these data can now be accessed at spatial and temporal scales similar to ecological field data (Kerr and Ostrovsky 2003).

### MODIS remotely sensed data

MODIS provides data with global coverage at 250 m^2^, 500 m^2^, and 1 km^2^ spatial resolutions collected twice daily by NASA's Terra and Aqua satellites since the year 2000. Raw data from the MODIS sensors are composited to daily, 8 day, 16 day, and yearly imagery and preprocessed into discipline-specific MODIS products for atmospheric, oceanic, or land process applications. The Land Processes Distributed Active Archive Center (LP DAAC – https://lpdaac.usgs.gov) holds MODIS data on metrics of land processes such as surface reflectance, land cover/land cover change, land surface temperature and emissivity, vegetation indices (Normalized Difference Vegetation Index, NDVI, and Enhanced Vegetation Index, EVI), leaf area index and fPAR (fraction of photosynthetically active radiation), evapotranspiration, net photosynthesis, and primary productivity (https://lpdaac.usgs.gov/products/modis_products_table; examples of their usage are shown in Table 1).

Many ecological studies, such as meta-analyses, observation networks or globally distributed networks of experiments, combine information from large numbers of locations. Such studies allow comparison of patterns across space and time and estimation of global responses. They can help to identify and understand generalities (Borer et al. 2014), and synthesize the literature to predict the responses of ecological communities to global change (Newbold et al. 2013). The global coverage of remotely sensed data presents a valuable resource for global ecological studies but the existing MODIS access mechanisms make it hard to obtain the relevant data.

Despite the increasingly common use of MODIS data, there is a burden on the investigator to access, download, and store them. The Oak Ridge National Laboratory Distributed Active Archive Center (ORNL DAAC) provides online access to spatially and temporally delimited subsets (ORNL DAAC 2014). The user can download one subset at a time, via email, after manually inputting subset definitions each time. Manual input of spatial and temporal limits can be both error-prone and time-consuming. A web service is available (http://daac.ornl.gov/MODIS/MODIS-menu/modis_webservice.html) that can facilitate automation but this requires familiarity with protocols and languages such as SOAP (Simple Object Access Protocol; Gudgin et al. 2007) and XML (Extensible Markup Language), which ecologists are typically not familiar with, are time-consuming to learn and shift attention away from science.

### Utilizing MODIS data with MODISTools

*MODISTools* – a package for the R Statistical Computing Language (R Core Team 2014) – allows MODIS data for multiple locations, time periods and products to be downloaded, and stored using a single line of R code. Downloaded data are stored in a simple, memory-efficient way that can be easily retrieved and manipulated. *MODISTools* provides functions for processing downloaded data and merging these data with the user's ecological response data, making it possible to apply MODIS data to research questions with minimal effort. By avoiding the time-consuming and often error-prone manual steps, the package simplifies access to MODIS data, thereby increasing research efficiency. *MODISTools* completes all functioning within the R environment without requiring external software.

We are aware of one other existing R package, *MODIS*, that downloads MODIS data (Mattiuzzi et al. 2013). This is geared toward use of data in a geographic information system (GIS), which often requires the user to download and interact with additional software, such as the MODIS Reprojection Tools (LP DAAC 2014). *MODISTools* completes all functioning within the R environment without requiring external software. The *MODIS* R package provides useful functions to download MODIS data at one or a few locations as raster files for use in GIS. Many users of MODIS data will require some data processing steps in a GIS environment, for example, extracting a complex shape, such as the boundary of a country or national park, from the downloaded rectangular MODIS tile. In our experience of ecological modeling, an ideal format for downloaded MODIS data has been in ASCII plain text format, which can be readily downloaded from the ORNL DAAC MODIS web service. Although *MODISTools* does not itself contain functions for GIS processing steps, it connects to a GIS environment efficiently by providing a function for creating ASCII raster grids from the downloaded files. These new files are provided with the correct MODIS projection datum (PRJ file) that allows them to be easily imported into standard GIS software.

### Example analysis

We demonstrate the utility of *MODISTools* applying MODIS data to ecological questions, particularly studies involving many sites, by conducting an illustrative analysis of our own. We analyzed species richness data from the literature, investigating the relationship between local species richness in temperate regions and spatiotemporally matched vegetation indices. We show every step from downloading data, through data processing, and analysis; this demonstrates the value that *MODISTools* provides in each step of the method and how it can aid researchers to apply MODIS data to ecological questions in general.

Many studies have reported a positive correlation between species richness and aboveground net primary productivity, although the relationship might not be linear depending on the spatial scale (Gaston 2000). Previous studies have established a relationship between aboveground net primary productivity and vegetation indices (Reed et al. 1994; Waring et al. 2006): vegetation indices describe the greenness of the vegetation and the Normalized Difference Vegetation Index (NDVI) is one of the most commonly used (Pettorelli et al. 2005). NDVI is calculated using red and near-infrared wavelengths of light that are reflected and captured by the satellite. Chlorophyll will absorb red light, while the mesophyll structure of a leaf will scatter reflected near-infrared light. Therefore, if the proportion of reflected near-infrared light captured is greater than red light captured this represents a signal of vegetation. NDVI values increase from 0 to 1 as the amount of vegetation increases, whilst negative values indicate an absence of vegetation (Myneni et al. 1995). An extension of NDVI, the Enhanced Vegetation Index (EVI), adjusts for atmospheric aerosol interference and improves sensitivity so values do not saturate in areas of high biomass (Huete et al. 2002). Because of their relationship with aboveground net primary productivity, NDVI and EVI are often used as predictors for species richness, with good results (Hurlbert and Haskell 2003; Seto et al. 2004; Waring et al. 2006). Time series, rather than one off measurements, of vegetation indices are required to capture vegetation dynamics relevant to the species richness data, and to minimize the contamination of this signal of vegetation dynamics due to noise.

Normalized Difference Vegetation Index is publicly available from various data sets; the earliest was collected from the National Oceanic and Atmospheric Administration's Advanced Very High Resolution Radiometer (NOAA-AVHRR), with data extending back to 1981 (James and Kalluri 1994). EVI has been proposed comparatively recently, but MODIS products provide global data for both indices. As with the multitude of other products available through the LP DAAC MODIS archive, these vegetation indices can be summarized in many ways to produce measures relevant to ecological study (see Pettorelli et al. 2005 for a review of NDVI measures).

**Figure 1:**
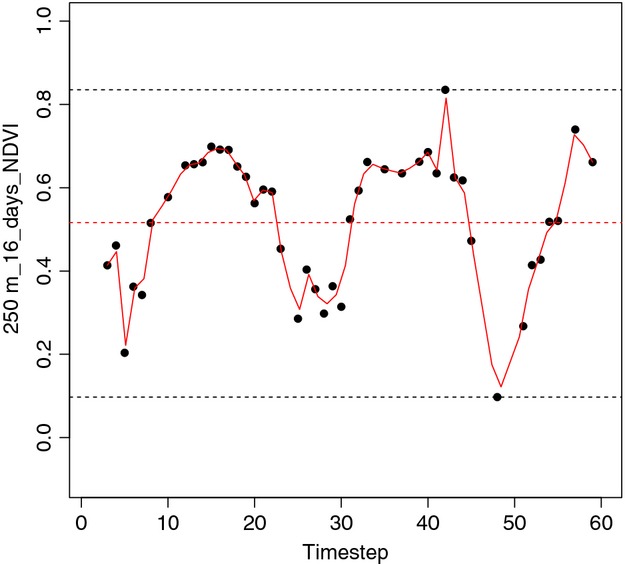
Time series of NDVI following a QualityScreen, as produced by an optional argument DiagnosticPlots in MODISSummaries, that illustrates the relationship between the three measures used: maximum time-series value, temporal mean, and temporal variability. On the y-axis is NDVI at 250 m^2^ resolution (the axis label is the data band name for this Science Data Set). On the x-axis is time, with 16-day regular intervals. This time series produces a temporal variability value of 0.4. Variability is defined in the introduction of the main text. Upper black dashed line indicates the maximum value in the time series and the lower black dashed line indicates the minimum value in the time series. The solid red line indicates the interpolated values of the NDVI. Red dashed line indicates the mean of these values.

Here, we focus on three measures of NDVI and EVI time series that are calculated by *MODISTools*: the maximum value in a time series, the temporal mean, and the temporal variability (Fig. 1). The time series were smoothed with temporal interpolation, using MODISSummaries, prior to calculating these summary statistics. Smoothing reduces noise in the time series due to cloud cover, or high solar or scan angles (Pettorelli et al. 2005). The temporal variability can be characterized as the average temporal variation of vegetation index within a site, factoring in the minimum value: it is the time-averaged difference between the total area under the time series and the area under the minimum value in the time series. Temporal variability is of interest when comparing among sites as the average variation in a vegetation index can be equal when the mean is not, and *vice versa*. In this context, temporal variability can be seen as a measure of seasonality; higher temporal variability indicates increased change in vegetation cover from one season to another. It could alternatively represent offtake, with a higher temporal variability indicating more harvesting at a site.

These summary measures of NDVI and EVI were the explanatory variables that we related to local species richness across temperate forests globally. The diversity data were sampled from the PREDICTS database (http://www.predicts.org.uk – Newbold et al. 2014; Hudson et al. unpublished data), a compilation of data from studies worldwide that have measured diversity (of one or more species) over multiple habitat types. Data were collected from published papers and, when necessary, subdivided further into studies with distinct methodologies. Within each study, there were multiple sites where diversity (in this case species richness) was measured. Our methods detail the utility that *MODISTools* provided at each stage. We then discuss our results and reflect on the benefits of using *MODISTools* for such research and more broadly. The code used to analyze the data and a dummy data set, generated from the real data we have analyzed, are available for reproducing the entire workflow presented (see Table S1, Appendices S1–S4).

## Method

### Data preparation

A subset of diversity data from the PREDICTS database was extracted using the following criteria: (1) studies were within the Temperate Broadleaf and Mixed Forests Biome (as determined by the WWF biome layer, Olson et al. 2001); (2) the studies sampling period ended post 2000 (to ensure MODIS data were available); (3) studies contained more than 20 sites; and (4) studies captured a diversity metric that could be converted to species richness. The resulting data set contained 526 sites from eight different studies (Brunet et al. 2011; Buczkowski 2010; Chapman and Reich 2007; Lantschner et al. 2008; Lentini et al. 2012; Su et al. 2011; Weller and Ganzhorn 2004; Winfree et al. 2007 – Magura et al. 2010 was also considered but removed from analysis due to small sample size).

Normalized Difference Vegetation Index and EVI were downloaded for each of the 526 sites using the MODISSubsets function. MODISSubsets fetches data from the ORNL DAAC MODIS Land Product Subsets web services. The input to the function is an R data.frame or a text file specifying all sites where diversity data were collected (ST1). For each site, this data frame must specify lat/long coordinates and dates for the time series requested. End dates and optionally start dates for each time series can be specified; or, just end dates can be given with an optional time-series length that MODISSubsets will consistently download for when the timespan of preceding data permits. Specific column names are necessary for MODISSubsets to find the information it needs: “lat,” “long,” “end.date,” and optionally “start.date”. The input coordinates must be in decimal degrees format with WGS84 datum. As with any spatial data, ensuring the coordinates consistently conform to the intended datum is imperative for correctly specifying locations. *MODISTools* provides the ConvertToDD function to reformat coordinates into decimal degrees from either degrees minutes seconds or degrees decimal minutes. The dates can be specified in two different formats: (1) years, in which case data will be downloaded from the beginning of the year, or (2) POSIX formatted dates (YYYY-MM-DD) that allow more precise requests.

Once the time series coordinates and dates have been specified, the user must delimit the area of interest around each location, defined as the distance in kilometers around each location. Two directions must be specified, firstly in the north/south direction from the focal pixel (pixel identified by the coordinate supplied), and secondly the east/west direction. Using the values (0,0) would result in data for the focal pixel only. The values (1,1) identify an area of interest that, in addition to the focal pixel, includes surrounding pixels for 1 km in each direction. The maximum area of interest surrounding a location that can be retrieved using the ORNL DAAC MODIS web service is (100,100). This two-dimensional input means rectangles of any size smaller than the maximum area of interest can be specified. The number of pixels included in a request depends on the resolution of the data requested: for NDVI data at 250 m^2^ resolution, a 2.25 × 2.25 km area, downloaded using values (1,1), will contain 81 pixels.

Users must also state what MODIS data product is required. Each data “product” released contains many variables that are stored in separate “Science Data Sets,” also known as data “bands.” For example, the EVI and NDVI bands that we require can be found in the vegetation indices product (MOD13Q1), alongside other variables and indicators of pixel reliability. Functions GetProducts and GetBands help specify the desired data type.

### Download the data

We downloaded NDVI and EVI at 250 m^2^ resolution as well as pixel reliability for these data to allow quality control before analysis. The size of area of interest used to relate vegetation indices to our diversity data may have an impact on the result. It may be that the most local conditions sufficiently explain species richness, or that the surrounding area must also be captured. We analyzed the data at both the focal pixel (Size = c(0,0)) and 6.25 × 6.25 km tile (Size = c(3,3)) scales and compared which best explained variation in species richness. The function ExtractTile can extract subsets from larger areas of interest to avoid duplicated downloads. To ensure all estimates are based on the same length of time series, we requested 3 years prior to the species richness sampling date at each site (year of sampling date plus TimeSeriesLength argument). The following line of code completes the download for the request described:

MODISSubsets(LoadDat = PREDICTS, Products =

“MOD13Q1”, Bands = c("250m_16_days_NDVI",

“250m_16_days_EVI”,

“250m_16_days_pixel_reliability”), Size = c(3,3),

StartDate = FALSE, TimeSeriesLength = 2)

For each site downloaded MODISSubsets saves a text file (ASCII format, comma separated, no header), with a log file listing all the unique downloads and their download status (see Table 2 for an explanation of downloaded data structure). The data saved to these files can be easily read back into R using MODISTimeSeries. If the input data set contains unique identifiers (IDs) for each site, these will be used as file names; if not, the function will generate unique IDs itself and use these. All files will be stored in a user-specified directory (working directory by default), and this directory path will be printed in R prior to downloading. The download speed primarily depends on server traffic at the ORNL DAAC MODIS web service, and to a lesser extent on internet connection and size of data set being downloaded. Speed can be highly variable for even the same data set. We replicated the download of our 526 sites and on one occasion it took 2–3 h and another approximately 12 h (see Table 3 for performance metrics).

**Table 2:** An explanation of the sections, for example, text string (in text), which shows the format of data subsets written in the ASCII text file outputs from MODISSubsets. These ASCII files can be read back into an R workspace using read.csv(“filename.asc”, header = FALSE, as.is = TRUE). The resulting data.frame would contain columns for each section described below and rows for each date in the time series

Section description	Example
Number of tile rows	1
Number of tile columns	1
*x*-coordinate (MODIS datum longitude) for lower left corner of tile	13702705
*y*-coordinate (MODIS datum latitude) for lower left corner of tile	–3709977
Pixel size (meters)	231.6564
Unique Identifier	MOD13Q1.A2009001.h30v12.005.2009020003129.250m_16_days_EVI
Shortname code for the MODIS product requested	MOD13Q1
Date code for this string of data, year and Julian day (A[YYYYDDD])	A2009001
Input coordinates and the width (Samp) and height (Line) in number of pixels of the tile surrounding the input coordinate	Lat-33.3636449991Lon147.548402Samp1Line1
Date–time that MODIS data product was processed (YYYYDDDHHMMSS)	2009020003129
All values following are data for each pixel in the tile (*n* = Samp × Line), which are ordered by row. The number of columns should equal the number of pixels in the tile – in this case 1. See ?ExtractTile to rearrange the data back into spatially ordered tiles.	1567

**Table 3:** Performance metrics for the downloading function, MODISSubsets, all times reported in seconds. The times reported here are for a simple subset request (one site, focal pixel only, for 1 year) using an example data.frame provided with *MODISTools* called SubsetExample. The effect of time-series length (3 years), tile size (2.25 × 2.25 km tile size – 81 pixels), and number of sites (four sites, using the *MODISTools* data.frame EndCoordinatesExample) on time taken to download is shown for multiple computers. The largest source of variation in download times will be traffic at the ORNL DAAC MODIS server, and internet connection

System	Simple request	Time-series length	Tile size	Number of sites
MacBook Air (2013)	51.962	119.687	32.177	173.033
Processor: 1.3 GHz Intel Core i5				
Memory: 8 GB 1600 MHz DDR3				
Software: OSX 10.9.4				
Internet: up to 30 MB wireless				
MacBook Pro	36.887	99.253	37.093	148.616
Processor: 2.4 GHz Intel Core 2 Duo				
Memory: 4 GB 1067 MHz DDR3				
Software: OSX 10.6.8				
Internet: up to 90 MB wireless				
MacBook Mini	47.387	112.134	39.210	158.606
Processor: 2.6 GHz Intel Core i7				
Memory: 16 GB 1600 MHz DDR3				
Software: OSX 10.8.5				
Internet: Network cable				

No internet service can guarantee 100% availability and MODISSubsets employs a strategy to work around temporary loss of availability of the ORNL DAAC MODIS server and to download as many of the requested subsets as possible. If MODISSubsets encounters a problem, such as a loss of connection, it produces a warning message and retries that subset until either it has been downloaded or 15 min have elapsed, after which time it attempts to download the next of the requested subsets. Any error and warning messages encountered during downloading are stored in the log file so they can be traced to the problematic time series. MODISSubsets then attempts a second download of any subsets that could not be fetched in the first pass, minimizing the amount of missing data in the final output. MODISSubsets writes each unique subset (i.e., combination of lat/long location and time-series start and end dates) to a single text file, minimizing download and storage load. The text files can easily be combined into a single text file (CSV format), along with the input ecological data, facilitating modeling (see Statistical analysis). UpdateSubsets can also be used to complete an unfinished download, or download for new sites added to a data set.

### Process the data

The MODISSummaries function collates the downloaded data and computes the per-pixel time-series summary statistics. Prior to summarizing the data, it uses the pixel reliability indicator to filter out poor-quality and missing data (data with pixel reliability values > 0 were omitted). MODISSummaries then assembles the pixel level summary statistics with the user's data (PREDICTS in our example) that contain response variables and returns a data frame that can be used for statistical modeling with existing R modeling tools. Hence using two function calls, one to download data (MODISSubsets function call above) and the other to process data (MODISSummaries function call below), we generated new columns in our ecological data set that provided the explanatory variables for our analysis:

MODISSummaries(LoadDat = PREDICTS, Product =

“MOD13Q1", Bands = c(“250m_16_days_NDVI”,

“250m_16_days_EVI”), ValidRange = c(-2000,10000),

NoDataFill = -3000, ScaleFactor = 0.0001,

StartDate = FALSE, QualityScreen = TRUE,

QualityBand = “250m_16_days_pixel_reliability”,

QualityThreshold = 0, Max = TRUE, Mean = TRUE,

Interpolate = TRUE, Yield = TRUE)

### Statistical analysis

We fitted generalized linear mixed effects models to the species richness data, using a poisson error distribution with a log link function as is appropriate for count data. To avoid collinearity among explanatory variables that are based on the same data (including EVI and NDVI variables, as EVI is an extension of NDVI), each variable was fitted in a different model. To account for homogeneity among sites within a study, we fitted the study grouping variable as a random effect. The fixed effects were the remotely sensed vegetation index measures, a higher taxon factor (Aves, Coleoptera, Hymenoptera, Pinopsida), and the interaction between these effects. Models were fitted using *lme4 v1.1-6* in R (Bates et al. 2014). The *R*^2^ for the models was calculated using the *R.squaredGLMM* function from the *MuMIn* R package (Barton 2014). *R*^2^ is a good indication of the goodness of fit of the data to the model and a simple indicator of how well the model could be used for prediction (Nakagawa and Schielzeth 2013).

## Results and Discussion

On average, species richness increased with increasing vegetation indices, as has been reported in previous studies (Waring et al. 2006). Our findings also corroborate previous results that have shown these responses to be taxa-specific (Bailey et al. 2004; Gibson et al. 2011; Newbold et al. 2013, 2014) and, although less frequent in our findings, scale-specific (Hurlbert and Haskell 2003). Overall, models at all scales produced similar *R*^2^: values range from 0.57 to 0.83, which suggests the models explain a large amount of variation in species richness and hold some predictive power. However, rather than being definitive results, these findings are preliminary and presented here primarily to highlight the use of *MODISTools*. Thus, only a subsection of the results are discussed in the text, but additional results and figures can be found in the Supporting Information (Appendices S3, S4, and Figs. S1, S2, S3).

**Figure 2:**
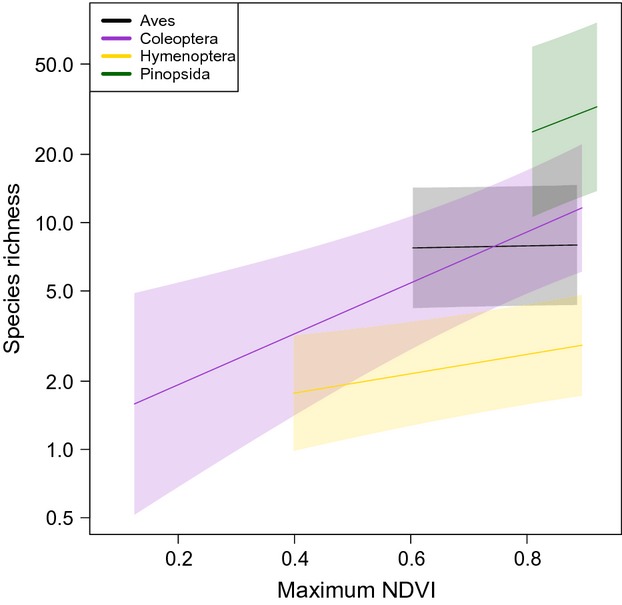
Responses of four taxa to changes in maximum NDVI. Aves (black line) showed no significant response to changes in maximum NDVI, while Coleoptera (purple line), Hymenoptera (yellow line) and Pinopsida (green line) showed a positive response to increasing maximum NDVI. Shaded areas indicate 95% confidence intervals.

Species richness among all higher taxa except Aves covaried positively with maximum NDVI at the focal pixel scale, but these responses were variable so the interaction term was retained (Fig. 2). The strongest relationships estimated were for Hymenoptera and Coleoptera. Although these slope estimates should be interpreted with caution, as our data set only included 22 sites for Coleoptera, previous studies have also shown strong positive correlations of beetle species to NDVI (Lassau and Hochuli 2008). Mean NDVI at both the small and large spatial scale showed a positive correlation with species richness for all taxa except Aves (Figs. S1 and S2). All higher taxa showed similar positive correlations with NDVI variability at the focal pixel scale (Fig. S1), but with varying intercepts; the interaction was retained, however, for NDVI variability at the larger scale.

In most cases, EVI and NDVI variables produced similar patterns, but the response of Pinopsida species richness to maximum value in a time series was dependent on the vegetation index used (Figs. S2 and S3). Unlike NDVI, EVI considers the nonlinear differences between the radiative transfer of red and near-infrared light through a canopy (Pettorelli et al. 2005). This may underlie the discrepancy between vegetation indices, as conifers produce a different tree architecture and canopy structure from deciduous trees. Leaf color may also be important: The bluish color found in adult trees of some species within Pinopsida would be incorporated into the EVI, which is calculated using blue reflectance, but not NDVI.

Despite previous work showing the contrary (see Koh et al. 2006; using 1 × 1 km NDVI derived from the SPOT-VGT imaging sensor), bird species richness was not significantly affected by changes in any of the vegetation index variables, at neither the small or large spatial scales. Seto et al. (2004) found that the relationship between bird species richness and vegetation index variables strengthened as the spatial extent considered increased, possibly due to birds having larger home ranges. Therefore, the lack of relationship we found within Aves may in part be caused by analyzing our remotely sensed variables at an inappropriate spatial extent.

## Conclusions

The benefits of the automated approach that *MODISTools* takes are valuable. All 526 subsets of data, for the entire timespan of interest, can be retrieved using one line of R code. Using any other method that cannot automate the process over subsets, such as the email service using the online tool, would require 526 independent subset requests to be input manually by the user: researcher time and opportunity for error would greatly increase. *MODISTools* provides a more scalable approach, so the benefit of using *MODISTools* increases with the number of subsets requested. Our tool was equally beneficial for the data processing steps (without using a GIS environment) by producing our MODIS derived variables and matching them to the species richness data, ready for ecological modeling.

The package is being actively developed, and future releases will include more product-specific post-download processing. Summaries suitable for vegetation indices, such as time-integrating vegetation indices, are already available but these will be expanded to include a range of summary functions for a wider set of MODIS products, for example, temporal Fourier processing (Scharlemann et al. 2008). Processing will become more flexible by allowing the user to write custom summary functions. Additional methods for quality control of time-series data will also be included, allowing interpolation of missing values using adjacent time-series data (Rowhani et al. 2008). The interaction between *MODISTools* and spatial processing tools, in R and GIS environments, can also be further expanded.

The current stable release of *MODISTools* is available on CRAN (http://cran.r-project.org/web/packages/) – the usual mechanism for making R packages readily available – and can be installed by running: install.packages(“MODISTools”). The latest “in-development” version of *MODISTools* can be accessed at https://github.com/seantuck12/MODISTools. For a more in-depth walkthrough to downloading and using MODIS data with *MODISTools*, see the associated vignette by installing the package and then entering vignette(“UsingMODISTools”) into R.

## Supporting Information

**Appendix S1.** R script for downloading MODIS vegetation indices for analysis.

**Appendix S2.** R function to create a vector of weighted means the same order as a vector.

**Appendix S3.** R script for analysing EVI and NDVI data for a single pixel.

**Appendix S4.** R script for analysing EVI and NDVI data for a 6×6 km area of interest.

**Figure S1.** Response of species richness to vegetation indices at the focal pixel scale.

**Figure S2.** Response of species richness to NDVI at the 6.25×6.25 km scale using spatially-weighted and unweighted data.

**Figure S3.** Response of species richness to EVI at the 6.25×6.25 km scale using spatially-weighted and unweighted data.

**Table S1.** Dummy ecological data set for conducting analysis.
