# Supplementary figures and images for: MODISTools – downloading and processing MODIS remotely sensed data in R
Source: Ecol Evol. 2014 Dec 2;4(24):4658–68. doi: 10.1002/ece3.1273 (PMC4278818; doi:10.1002/ece3.1273)

Figure SA5. Response of species richness to vegetation indices at the focal pixel scale

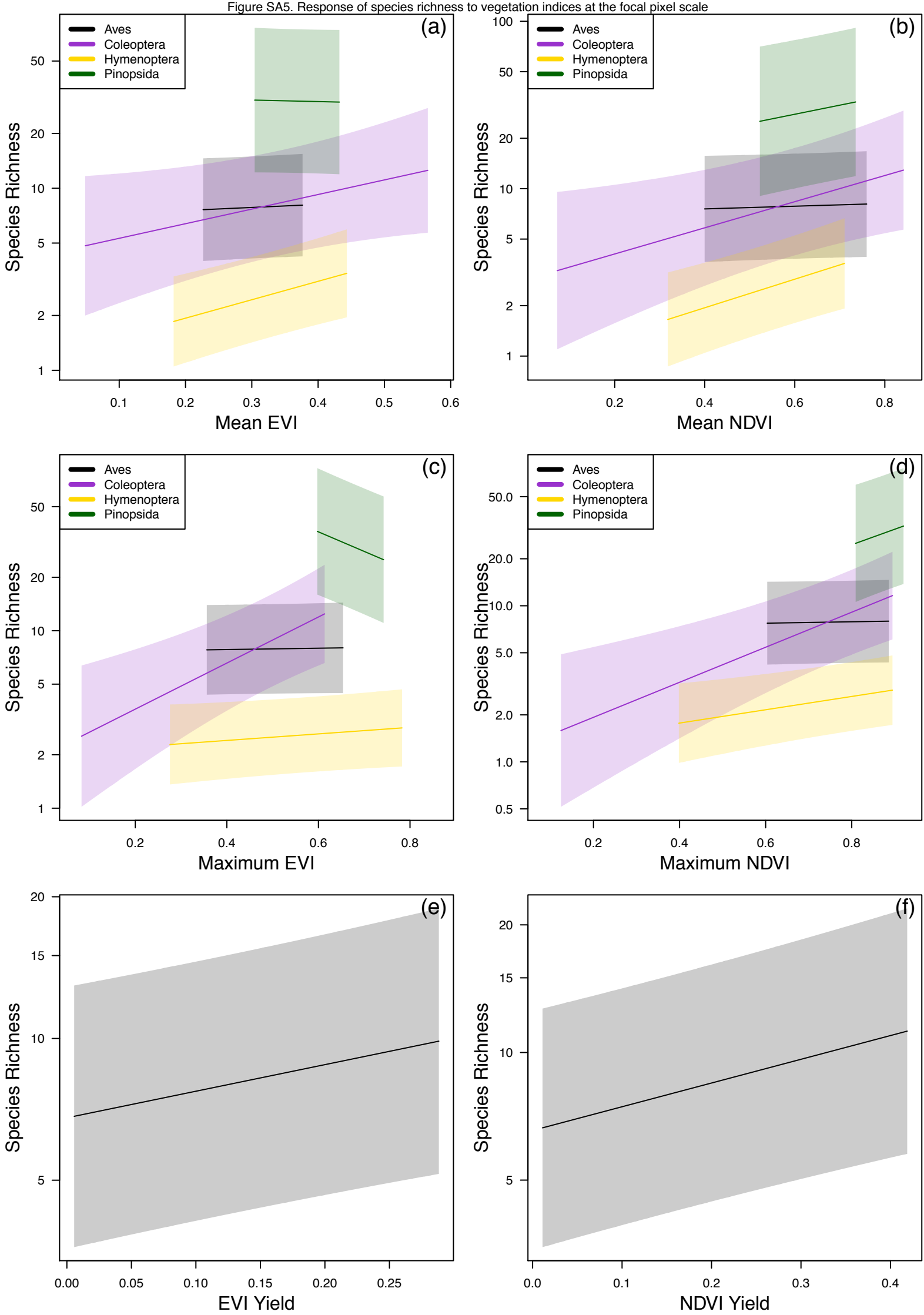

Supplement: Supplementary file 5 — Figure S1. Response of species richness to vegetation indices at the focal pixel scale. [file ece30004-4658-sd5.pdf]
