# Supplementary material for: MODISTools – downloading and processing MODIS remotely sensed data in R
Source: Ecol Evol. 2014 Dec 2;4(24):4658–68. doi: 10.1002/ece3.1273 (PMC4278818; doi:10.1002/ece3.1273)

Figure SA6. Response of species richness to NDVI at the 6.25x6.25 km scale using spatially-weighted and unweighted data

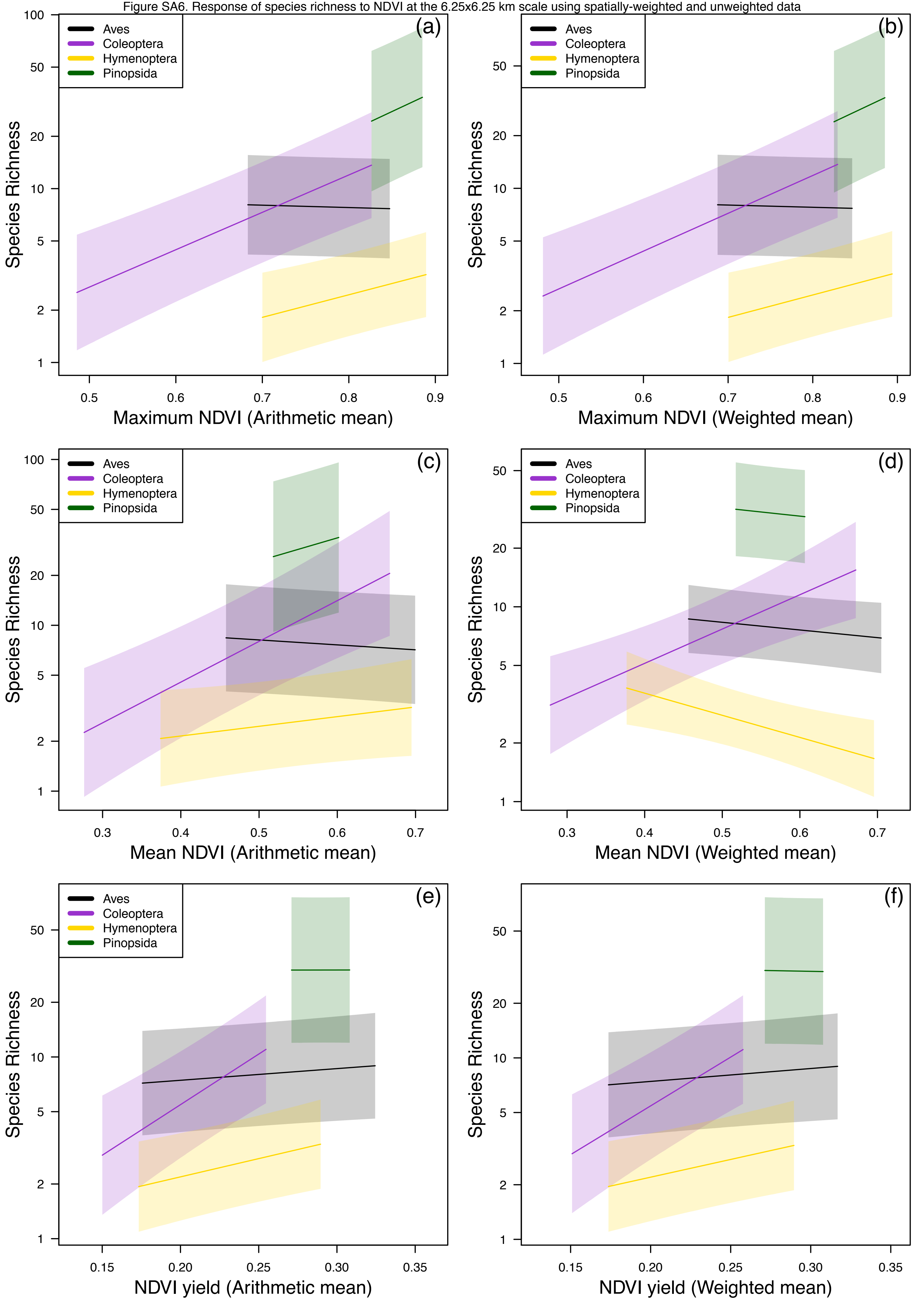

Supplement: Supplementary file 6 — Figure S2. Response of species richness to NDVI at the 6.25×6.25 km scale using spatially-weighted and unweighted data. [file ece30004-4658-sd6.pdf]
